# CardioTox net: a robust predictor for hERG channel blockade based on deep learning meta-feature ensembles

**DOI:** 10.1186/s13321-021-00541-z

**Published:** 2021-08-16

**Authors:** Abdul Karim, Matthew Lee, Thomas Balle, Abdul Sattar

**Affiliations:** 1 School of Information Communication Technology, Griffith University, 4111 Nathan, Brisbane, Australia; 2 Sydney Pharmacy School, Faculty of Medicine and Health, The University of Sydney, 2006 Sydney, Australia; 3 Brain and Mind Centre, The University of Sydney, 2050 Sydney, Australia; 4 Institute of Integrated and Intelligent Systems, Griffith University, 4111 Nathan, Brisbane, Australia

**Keywords:** Deep Learning, Meta ensembling, Meta features, Cardiotoxicity

## Abstract

**Motivation:**

Ether-a-go-go-related gene (hERG) channel blockade by small molecules is a big concern during drug development in the pharmaceutical industry. Blockade of hERG channels may cause prolonged QT intervals that potentially could lead to cardiotoxicity. Various in-silico techniques including deep learning models are widely used to screen out small molecules with potential hERG related toxicity. Most of the published deep learning methods utilize a single type of features which might restrict their performance. Methods based on more than one type of features such as DeepHIT struggle with the aggregation of extracted information. DeepHIT shows better performance when evaluated against one or two accuracy metrics such as negative predictive value (NPV) and sensitivity (SEN) but struggle when evaluated against others such as Matthew correlation coefficient (MCC), accuracy (ACC), positive predictive value (PPV) and specificity (SPE). Therefore, there is a need for a method that can efficiently aggregate information gathered from models based on different chemical representations and boost hERG toxicity prediction over a range of performance metrics.

**Results:**

In this paper, we propose a deep learning framework based on step-wise training to predict hERG channel blocking activity of small molecules. Our approach utilizes five individual deep learning base models with their respective base features and a separate neural network to combine the outputs of the five base models. By using three external independent test sets with potency activity of IC_50_ at a threshold of 10 $\upmu$m, our method achieves better performance for a combination of classification metrics. We also investigate the effective aggregation of chemical information extracted for robust hERG activity prediction. In summary, CardioTox net can serve as a robust tool for screening small molecules for hERG channel blockade in drug discovery pipelines and performs better than previously reported methods on a range of classification metrics.

**Supplementary Information:**

The online version contains supplementary material available at 10.1186/s13321-021-00541-z.

## Background

The human ether-à-go-go-related gene (hERG) encodes a voltage-dependent ion channel (Kv11.1, hERG) involved in controlling the electrical activity of the heart by mediating the re-polarisation current in the cardiac action potential [1, 2]. Malfunction or inhibition of hERG-channel activity by drug molecules can lead to cardiac arrhythmias in the form of prolonged QT intervals and may lead to sudden cardiac arrest. Therefore, unwanted drug-induced arrhythmias are great concern for pharmaceutical companies and have led to blockbuster drugs being withdrawn from the market and discontinuation of drugs in late stages of development [3]. To prevent new drugs with unwanted hERG-related cardiotoxicity to enter the market, guidelines for assessment of potential for QT interval prolongation by non-cardiovascular medicinal products were decided at the International Conference on Harmonization of Technical Requirements for the Registration of Pharmaceuticals for Human Use (ICH) [4, 5]. These procedures are time-consuming and expensive and therefore, to prevent product depletion due to cardiotoxicity at late preclinical and clinical stages, there is focus on preventing drugs with hERG channel activity from entering drug discovery pipelines in the first instance. To avoid this, computational methods to predict hERG liability have been established and can help prioritise molecules during the early phase of drug development [4]. Most of these methods are based on either machine learning techniques, including random forest (RF), support vector machine (SVM), deep neural networks (DNN) and graph convolutional neural networks (GCN) or on structure based methods including pharmacophore searching, quantitative structure activity relationships (QSAR) and molecular docking [6–10]. Publicly available high quality datasets consisting of molecules classified as hERG and non-hERG blockers are available and often utilized by these computational tools [6, 8, 11]. The datasets annotate chemical structure by SMILES strings which is a chemical language that describes the chemical structure using ASCII character strings. The SMILES strings are readable by expert chemists and are considered a low-level representation of molecular structure [12]. For ease of computational processing, chemical structure is encoded using a fragmentation scheme into binary vectors of fixed length called fingerprints which is another low level representation [13, 14]. Similarly, high level features such as 2D and 3D physicochemical descriptors can be computed from SMILES strings which are then used in various machine learning models [8, 15]. Alternatively, molecular graph representations have been used with graph convolutional neural networks [16]. This intermediate level molecular graph representation offers a compromise between high level physicochemical features and low level SMILES and fingerprints [17]. Under this category, each molecule can be represented via a molecular graph which consists of node features and an adjacency matrix.

Models in most of these previous studies utilize single type of features such physicochemical, fingerprints or graph features which restricts the model performance and its robustness [6, 8, 11, 18]. For instance, CardPred used a total of 3456 physicochemical descriptors and fingerprints with six individual machine learning models [8] to achieve reasonable performance when evaluated against accuracy (ACC) and positive predictive value (PPV) but performed poorly when evaluated against other metrics such as Matthew correlation coefficient (MCC), negative predictive value (NPV), specificity (SPE), sensitivity (SEN) (evaluated on external test sets as reported in the results section) [19]. A method reported by Cai et al. [6] relies on physicochemical descriptors and molecular vectors combined together as a single input for a fully connected multi-task deep neural network to achieve better performance for various metrics except NPV (for their internal cross validation datasets). Li et al. [11] used 8 different types of machine learning models and their ensemble with physicochemical descriptors and fingerprints performed well when evaluated against SPE and PPV but less so for other metrics. The key to success for these previous methods for hERG activity prediction is elucidating correct structure-property relationships from existing data using high level physicochemical features along with fingerprints. Recently the DeepHIT method was introduced which utilizes physicochemical descriptors, fingerprints and graph features with fully connected deep neural networks and graph convolution neural networks to achieving better performance for hERG activity prediction [19]. DeepHIT classifies a molecule as a hERG blocker if at least one model out of the three models used predicts a given molecule as a hERG blocker [19], thus enhancing the sensitivity of the model. Although DeepHIT utilize reasonably diverse feature set, it still lacks in an effective way of combining the outputs of individual models for robust performance over a range of metrics. There is also substantial literature for combining various types of features and features selection for molecular activity prediction, but no clear winner is concluded as yet because performance depends on the characteristics of the molecules used for modeling [20]. In several cases though, it was observed that the accuracy of the models can be improved by feature aggregation because of complementary information [20–23].

We hypothesize that extraction of chemical information from all or the subsets of three levels of features (low, high and intermediate) and their variants can improve upon the performance over a wide range of accuracy metrics for molecular hERG activity prediction For this purpose, we propose a step-wise training based deep learning framework called CardioTox net, that improves upon the previously published best-in-class results in most of the performance metrics. For three different external test sets, CardioTox net improves Matthew correlation coefficient with a value of (0.599, 0.452, 0.220), accuracy (0.810, 0.755, 0.746), positive predictive value (0.893, 0.455, 0.113) and specificity (0.786, 0.600, 0.698) while keeping the sensitivity same as so far the second best in class method, DeepHIT. Our framework consists of three stages; a featurization stage which generates base features; an individual prediction stage which uses base features with the base individual deep learning models to generate the outputs also called meta features; and a meta ensemble stage which uses meta features generated by the previous stage to classify the molecule as hERG blocker or hERG non-blocker.

## Materials and methods

### Data preparation

A dataset consisting of molecular structures labelled as hERG and non-hERG blockers in the form of SMILES strings was obtained from the DeepHIT authors [19] and was curated from five sources, the BindingDB database (3056 hERG blockers, 3039 hERG non-blockers) [24], ChEMBL bioactivity database (4859 hERG blockers, 4751 hERG non-blockers) [25], and literature derived (4355 hERG blockers, 3534 hERG non-blockers) [6], (1545 hERG blockers, 816 hERG non-blockers) [7], (2849 hERG blockers, 1202 hERG non-blockers) [26] and unlike in the DeepHIT procedure, we did not use any in-house data. A total of 30000 molecular structures were obtained and were standardized using RDkit [27] and MolVS [28] as described by Ryu et al. [19]. We further removed inconsistently labeled compounds. Thus we obtained total of 12620 molecules with 6643 labelled as hERG blockers and 5977 as hERG non-blockers to constitute our training set. We evaluated our framework against two external independent test sets, one of which was obtained from the authors of DeepHIT [19], hereafter called test-set I which is positively imbalanced (i.e. more blockers (30) than non-blockers (14)). We also retrieved other two independent test sets, thereafter called test-set II from [29, 30] and test set III from [31] as per the criteria of half maximal inhibitory concentration (IC_50_) values $< 10\, \upmu \hbox {M}$ considered to be hERG blockers and (IC_50_) values $\ge 10\,\upmu \hbox {M}$ considered to be hERG non-blockers. Test-set II is relatively smaller with 11 blockers and 30 non-blockers whereas Test-set III is relatively larger with 53 blockers and 786 non-blockers. The Tanimoto similarity [19] criteria was also ensured for all molecules in both test and training sets (explained in upcoming section of similarity and chemical diversity). The training set was subdivided into four sets, 70% for training the base models, 10% for validating base models, 10% for training the meta ensemble model and 10% for validating the meta ensemble model. *The detailed process of data preparation is given in * Additional file 1: S1. It should be noted that all the three independent data sets are imbalanced with higher number of hERG non-blockers. As per our knowledge at the time of conducting this research, these are most of the molecules available in public repositories which are dissimilar to our training data. This also demonstrates the real-world scenario for testing where number of non-blockers is usually more than the number of blockers.

### Similarity and chemical diversity

**Fig. 1:**
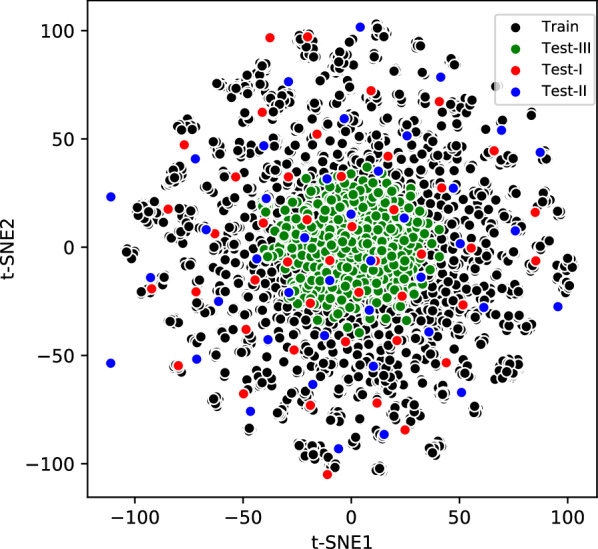
Two dimensional t-SNE components showing the chemical space diversity of training and the three external test sets

**Fig. 2:**
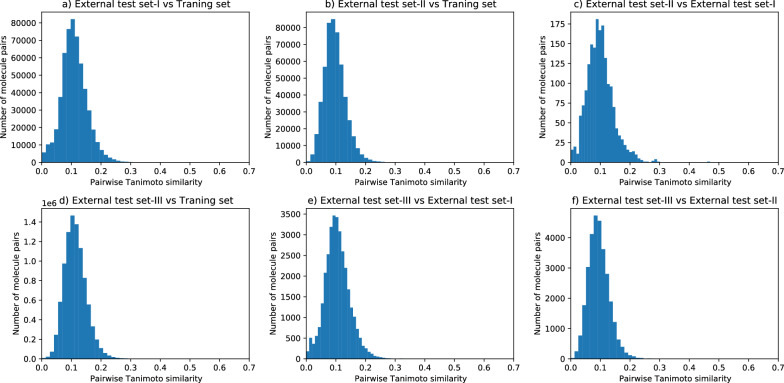
Pairwise Tanimoto similarity for each molecule in (**a**) external test-set I with all molecules in training set. **b** external test-set II with all molecules in training set. **c** external test-set I with all molecules in external test-set II. **d** external test-set III with all molecules in training set. **e** external test-set III with all molecules in external test-set I. **f** external test-set III with all molecules in external test-set II

A diverse dataset covering a broad chemical space is a prerequisite for building predictive models [32]. For all SMILES strings in training as well as in both external test sets, we computed the 2048 bit Morgan fingerprints using RDKit [13]. The t-SNE dimensional reduction technique [33] was then used to convert the 2048 dimensional vector into two t-SNE dimensions for each SMILES string. As demonstrated by the chemical space defined by the t-SNE components in Fig. 1, diverse chemical space distributions for classified blockers and non-blockers as well as overlap with the external tests sets was observed. We computed the Tanimoto mean value for each of the datasets separately given in Table 1 and a pairwise Tanimoto similarity shown in Fig. 2 for all four datasets [13]. The Tanimoto mean value shows the mean Tanimoto similarity within each data set whereas pairwise Tanimoto similarity shows similarity between different datasets. The lower the Tanimoto mean value is, the better the diversity of the compounds within the data set. As illustrated in Table 1, the Tanimoto mean value is 0.124 for the training set, 0.126 for the external test-set I, 0.116 for the external test-set II and 0.115 for the external test-set III, which means all the three data sets are diverse. Pairwise Tanimoto similarity as shown in Fig. 2 for external test sets, with respect to the training set is always less than 0.7. The external test-set I is also substantially dissimilar to the external test-set II as the maximum pairwise Tanimoto similarity value is less than 0.5 as shown in Fig. 2c. Similarly, we can see that external test-set III is also dissimilar to the training and other test-set-I and test set-II. We also provide top 3 more similar molecules in training data for each molecules of all three test sets in Additional file 3.

**Table 1:** Statistical description of data sets

Data set	Activity	Threshold	hERG blockers	hERG non-blockers	Total	Tanimoto mean
Training set	IC_50_	$10 \,\upmu \hbox {M}$	6643	5977	12620	0.124
Test set-I	IC_50_	$10 \,\upmu \hbox {M}$	30	14	44	0.136
Test set-II	IC_50_	$10 \,\upmu \hbox {M}$	11	30	41	0.116
Test set-III	IC_50_	$10 \,\upmu \hbox {M}$	30	710	740	0.115

### Evaluation criteria

In order to measure the classification performance of CardioTox net, we used the following metrics: Area under curve of receiver operating curve (AUC-ROC), specificity (SPE), sensitivity (SEN), negative predictive value (NPV), positive predictive value (PPV), accuracy (ACC) and Matthew’s correlation coefficient (MCC). The details of these metrics are as follows:

- *Area under curve of receiver operating curve (AUC-ROC)* which takes into account all the thresholds. The higher the value of AUC-ROC, the better the model is distinguishing between classes (hERG blockers and hERG non blockers). It can be computed by taking area under the curve for true positive rate (TPR) on the y-axis and false positive rate (FPR) on the x-axis for a given dataset. It should be noted that positive refers to hERG blocker and negative refers to non-hERG blocker. TPR which is also called sensitivity (SEN) describes how good the model is at classifying a molecule as a hERG blocker when the actual outcome is also a hERG blocker. FPR describes how often a hERG blocker class is predicted when the actual outcome is non-hERG blocker.
  1 $$\begin{aligned} SEN= & {} TPR = \frac{TP}{TP + FN} \end{aligned}$$
  2 $$\begin{aligned} FPR= & {} \frac{FP}{FP + TN} \end{aligned}$$
  where TP = True Positives, TN = True Negatives, FP = False Positives, and FN = False Negatives, SEN = Sensitivity.
- *Specificity (SPE)* is the total number of true negatives divided by the sum of the number of true negatives and false positives. Specificity would describe what proportion of the non-hERG blocker class got correctly classified by our model.
  3 $$\begin{aligned} SPE = \frac{TN}{TN + FP} \end{aligned}$$
- *Negative predictive value (NPV)* describes the probability of a molecule predicted as non-hERG blocker to be actually as non-hERG blocker.
  4 $$\begin{aligned} NPV = \frac{TN}{TN + FN} \end{aligned}$$
- *Positive predictive value (PPV)* describes the probability of a molecule predicted as hERG blocker to be actually as hERG blocker.
  5 $$\begin{aligned} PPV = \frac{TP}{TP + FP} \end{aligned}$$
- *Accuracy (ACC)* is the fraction of prediction our model got right. i.e it predicted hERG blocker and non-hERG blocker correctly.
  6 $$\begin{aligned} ACC = \frac{TP + TN}{TP + TN + FP + FN} \end{aligned}$$
- *Matthews Correlation Coefficient (MCC)* has a range of −1 to 1 where −1 indicates a completely wrong binary classifier while 1 indicates a completely correct binary classifier.
  7 $$\begin{aligned} MCC = \frac{TP * TN - FP * FN}{\sqrt{(TP + FP)(TP+FN)(TN+FP)(TN+FN)}} \end{aligned}$$

### Featurization stage

**Fig. 3:**
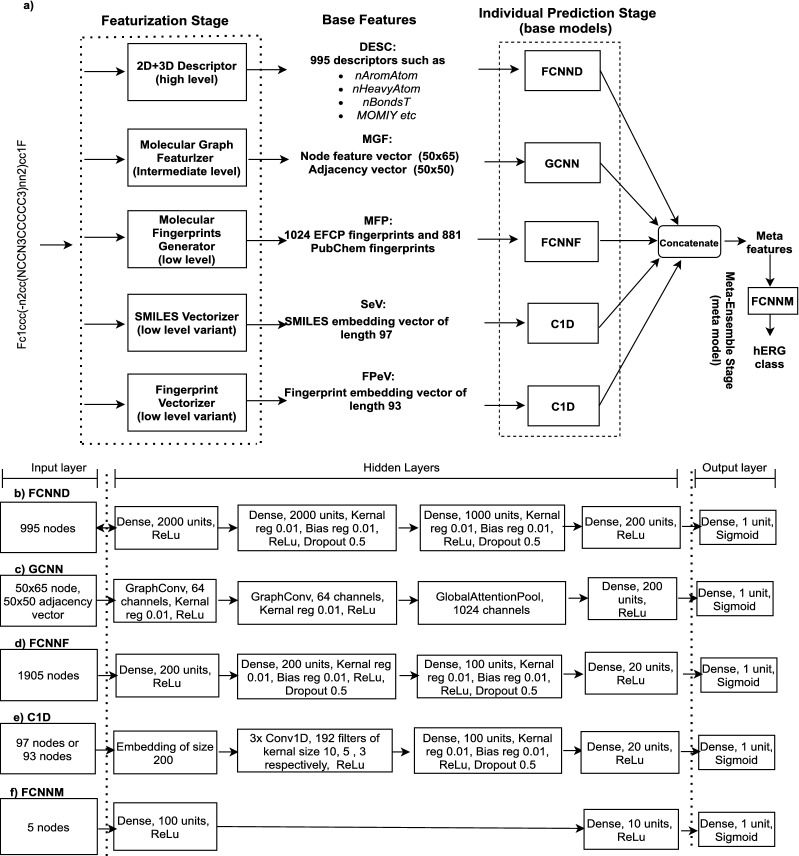
**a** CardioTox framework: End to end flow diagram of all the stages of proposed framework. **b** Architecture specifications of fully connected neural network for 995 2D and 3D descriptors as base features. **c** Architecture specifications of graph convolutional neural network for node vector of size 50x65 and adjacency vector of size 50x50 as base features. **d** Architecture specifications of fully connected neural network for 1024 EFCP and 881 pubchem fingerprints as base features (**e**) Architecture specifications of 1D convolution neural network for SMILES and fingerprints embedding vectors as base features. **f** Architecture specifications of meta ensemble fully connected neural network for meta features

The featurization stage of our framework consists of various types of featurizers which takes SMILES string as an input and produce fixed length base features as shown in Fig. 3a.

#### Descriptors

A total of 995 high level features such as 2D and 3D physicochemical descriptors (DESC) were computed using Mordred [34], names of which are also given in Additional file 2: S5. These features are numerical in nature and describe the physical and chemical properties of molecules [35]. 2D descriptors represents information related to size, shape, distribution of electrons, octanol-water distribution coefficient (LogP) which is a measure for lipophilicity, nAromAtom which shows number of aromatic atoms, nHeavyAtom which shows number of heavy atoms, nBondsT shows number of triple bonds. 3D descriptors relates to the 3D conformation of the molecules such as moment of inertia along Y axis (MOMIY) [35]. The value of each descriptor was normalized between 0 and 1.

#### Molecular graph featurizer

Topological information of molecules can be intuitively and concisely expressed via molecular graph features. This intermediate level featurizer computes molecular graph features such as node vectors which represents atoms in the SMILES string and an adjacency matrix which shows the bonds between atoms [17]. In this study, we extracted the same graph features as were extracted for DeepHIT [19], i.e a [50 × 65] node vector and a [50 × 50] adjacency matrix, details of which are also given in Additional file 2: S6. Here 50 refers to the maximum number of atoms and 65 refers to the one hot-encoded feature vector computed from atom descriptors [19].

#### Molecular fingerprint generator

The third featurizer deals with fingerprints where structural features are represented by either bits in a bit string or counts in a count vector [36, 37]. 1024 extended-connectivity fingerprints with a maximum diameter parameter of 2 (EFCP2) fingerprints and 881 pubchem fingerprints were computed using using the Python package PyBioMed [19, 38]. EFCP are also referred to as circular fingerprints and are specifically designed for structure-activity relationship modeling [39] whereas pubchem fingerprints are mainly designed for similarity neighboring and similarity searching [40].

#### SMILES vectorizer

We also computed two variants of low level features, SMILES strings embedded vectors (SeV) [41, 42] and fingerprint based embedded vectors (FPeV) [14] which themselves do no directly describe any biological attribute of the molecules, but has proven to have a reasonable predictive power in various quantitative structure-activity relationship (QSAR) tasks. In the SMILES vectorizer, we created a vocabulary based on the valid SMILES tokens (procedure described in Additional file 1: S2). A total of 64 unique tokens were determined based on the training data. The longest SMILES string in the data considered for this study was 97. Each SMILES string was converted into a one-hot encoded vector based on the SMILES vocabulary.

#### Fingerprints vectorizer

In the fingerprint vectorizer, SMILES string are converted into 1024 bit Morgan (or circular) fingerprints with a radius of 2 via RDKit [13]. As per the previously published technique [14], we extracted fingerprint indices which were marked 1 in the fingerprint generated. Thus we obtained a vector of length 93 which consisted of integers representing presence of specific substructures in a molecule. The procedure for fingerprint embedding vector is described in Fig. 1 of FP2VEC [14].

### Individual prediction stage

The individual prediction stage consists of base models which are trained on respective base features from the featurization stage. All of the base models were trained at a learning rate of $10e^{-4}$ with an Adam optimizer and 100 epochs with a batch size of 32. Selection of parameters, hyper-parameters and network architecture of base models were inspired from the previous published research in this area [8, 14, 15, 19, 41–43]. Each of these base models produce an output which is a single probability of a molecule being a hERG blocker. Here we describe each base model in the individual prediction stage also shown in Fig. 3b–e. The Keras deep learning framework and Spektral package was used in developing base models for the individual prediction stages [44, 45].

#### Fully Connected Neural Network for Descriptors (FCNND)

A fully connected deep neural network with 4 hidden layers was trained and validated on 995 2D and 3D physicochemical descriptors. The input layer consists of 995 nodes as per the number of total physicochemical descriptors and an output layer with 1 unit. All the layers in FCNND are densely connected and receives input from all the units present in the previous layer. The number of units in each hidden layer is decreased gradually and a ReLu activation [46, 47] is applied at the end of each layer. Kernel regularizer and bias regularizer of values 0.01 were used in training [47, 48] to reduce the over-fitting during optimization. Kernel regularizer applies penalties to the Kernel (main units in layer) and bias regularizer applies penalties to the bias units. We also applied a drop-out rate of 0.5 to the middle layers [49].

#### Graph Convolutional Neural Network for Graph features (GCNN)

A graph convolutional neural network (GCNN) was trained using the graph features as shown in Fig. 3c. GCNN consists of two graph convolution layers [50], one global attention pool layer [51] and a dense layer before the output. Each of the graph convolutional layers were initiated with 64 channels with a Kernel regularization value of 0.01 and a ReLu activation. The number of channels in the global attention pool layer was made equal to the number of units in the following dense layer, i.e 1024.

#### Fully Connected Neural Network for Fingerprints (FCNNF)

A fully connected neural network was used with fingerprints (FCNNF) as the base feature. Unlike FCNND, FCNNF uses a much smaller number of units in each layer. Except the number of units, other parameters were kept the same as in FCNND. The number of input nodes in the input layer were kept at 1905 to match the sum of 1024 EFCP fingerprints and 881 pubchem fingerprints as shown in part Fig. 3d.

#### Convolution 1D Neural Network for SMILES and Fingerprint embedding vectors (C1D)

For models where SMILES and fingerprint embedding vectors were used as base features, we used a variant of a Convolution 1D Neural Network (C1D) as base model as shown in Fig. 3e. The only difference was in the number of input-layer nodes which was 97 for SMILES embedding vectors and 93 for fingerprint embedding vectors. Input vectors were converted to a trainable embedding matrix of the size [97 or 93 × 200] which was then fed into a series of three 1D convolution layers. Each of these 1D convolution layers used ReLu activation, 192 filters with a Kernel size of 10, 5 and 3 respectively. Two densely connected layers with the parameters shown in Fig. 3e are also used to before the output layer.

### Meta ensemble stage

The outputs of each of the base models in the individual prediction stage were concatenated to produce meta features for the meta ensemble model. The Meta ensemble model is a fully connected neural network (FCNNM) with an input, output and two hidden layers as shown in Fig. 3f. It is trained at a learning rate of $10e^{-3}$ with an Adam optimizer and 300 epochs with a batch size of 32.

## Results and discussion

Our proposed framework employs step-wise training to produce the final classification of molecules as hERG or non-hERG blockers. For this purpose, data was divided into four sets, base training set: 70% for training base models , base validation set: 10% for validating base models, meta training set: 10% for training meta-ensemble model and meta validation set: 10% for validating the meta-ensemble model. In the first step of training, all the base models were trained on the base training set and validated using the base validation set. In the second step, the outputs of the best performing base models for the base validation set were used as meta features to train the meta ensemble model with the meta training set. We used the meta validation set to obtain the best meta ensemble model and also to select which combination of the base models ensembling produces better results. We performed consecutive splitting 10 fold cross validation [52] to obtain results given in the following subsection. For each time, we divided the data into 10 parts. Seven parts were used for base training, one part for base validation, one part for meta training and one part for meta validation.

### Validation of base model performance

The 10 fold cross validated results for individual base models of our framework on base validation set are shown in Table 2. Each base model is trained and validated with its own respective base features independently. In the Table 2, DESC refers to high level features such as 2D and 3D descriptors feeding the FCNND, MGF refers to intermediate molecular graph features fed into GCNN, MFP refers to low level molecular fingerprints fed into FCNNF, SeV refers to one of the low level variant i.e, SMILES embedding vectors when used with C1D and FPeV refers to low level variant i.e, fingerprint embedding vectors when used with C1D.

**Table 2:** 10 fold cross validated performance of the base models in individual prediction stage on base valid set using their respective base features

Base features	MCC	NPV	ACC	PPV	SPE	SEN	AUC
DESC	0.689	0.813	0.845	0.870	0.868	0.822	0.911
MGF	0.620	0.805	0.810	0.817	0.794	0.826	0.888
MFP	0.683	0.830	0.841	0.855	0.837	0.845	0.915
FPeV	0.638	0.814	0.818	0.826	0.802	0.835	0.899
SeV	0.636	0.811	0.817	0.827	0.809	0.826	0.889

Standard deviation value for each split for the above table is given in Additional file 1: S3

Highest values are underlined

As shown in Table 2, DESC performed better in MCC, ACC and PPV whereas MFP performed better in NPV, SEN and AUC. The possible reason might be the direct biological relevance of these base features (descriptors and fingerprints) to the activity prediction. Interestingly, SeV and FPeV showed better performance than MGF despite no biological relevance of the features used. FPeV and SeV achieved almost similar performance in most the of performance metrics. MGF legs behind in most of the metrics except SEN where it achieved slightly better performance than DESC.

### Meta validation performance

The overall goal of this study is to aggregate the chemical information extracted from various base features for cardio-toxicity data set so that the classification performance can be improved over a wide range of metrics. For that purpose, the outputs of the base models are concatenated to produce meta features for the use of a meta ensemble model as shown in Fig. 3a. A separate meta training set and meta validation set is used for training and validating the meta ensemble model. Table 3 demonstrates 10 fold cross validation results for the meta validation set for ensembling all possible unique combinations of base features ranging from 1 to 5. For instance, M1 represents single type of base features used in creating meta features whereas M2, M3, M4 and M5 represents any two, three, four and 5 different types of the base features with no repetitions.

**Table 3:** 10 fold cross validation results for various meta features on meta validation set

Meta Features	Base features	MCC	NPV	ACC	PPV	SPE	SEN	AUC
M1-1	DESC, DESC	0.676	0.829	0.838	0.862	**0**.**868**	0.819	0.909
M1-2	MGF, MGF	0.599	0.784	0.799	0.815	0.792	0.806	0.878
M1-3	MFP, MFP	0.682	0.829	0.840	0.853	0.838	0.843	0.909
M1-4	FPeV, FPeV	0.636	0.820	0.817	0.819	0.795	0.839	0.897
M1-5	SeV, SeV	0.621	0.806	0.809	0.816	0.791	0.828	0.880
M2-1	MGF, MFP	0.691	0.826	0.846	0.864	0.850	0.842	0.919
M2-2	MGF, DESC	0.683	0.818	0.842	0.865	0.848	0.835	0.914
M2-3	MGF, SeV	0.685	0.837	0.842	0.848	0.830	0.854	0.916
M2-4	MGF, FPeV	0.682	0.828	0.841	0.854	0.833	0.848	0.916
M2-5	MFP, DESC	0.710	0.843	0.855	0.866	0.855	0.855	0.928
M2-6	MFP, SeV	0.698	0.838	0.849	0.861	0.844	0.853	0.921
M2-7	MFP, FPeV	0.690	0.831	0.845	0.859	0.840	0.850	0.920
M2-8	DESC, SeV	0.707	0.847	0.853	0.860	0.846	0.861	0.926
M2-9	DESC, FPeV	0.715	0.848	0.857	0.867	0.859	0.856	0.929
M2-10	SeV, FPeV	0.680	0.828	0.840	0.853	0.835	0.845	0.918
M3-1	MGF, MFP, DESC	0.707	0.851	0.853	0.857	0.841	0.866	0.924
M3-2	MGF, MFP, SeV	0.711	**0**.**855**	0.855	0.857	0.835	**0**.**874**	0.927
M3-3	MGF, MFP, FPeV	0.701	0.849	0.850	0.853	0.833	0.867	0.921
M3-4	MGF, DESC, SeV	0.710	0.847	0.855	0.864	0.849	0.861	0.926
M3-5	MGF, DESC, FPeV	0.706	0.853	0.852	0.855	0.831	**0**.**874**	0.928
M3-6	MGF, SeV, FPeV	0.697	0.844	0.849	0.854	0.838	0.859	0.925
M3-7	MFP, DESC, SeV	0.718	0.854	0.859	0.865	0.850	0.868	**0**.**930**
M3-8	MFP, DESC, FPeV	0.710	0.850	0.855	0.861	0.846	0.864	0.926
M3-9	MFP, SeV, FPeV	0.699	0.837	0.849	0.862	0.848	0.851	0.925
M3-10	DESC, SeV, FPeV	0.712	0.846	0.856	0.866	0.854	0.858	0.928
M4-1	MGF, MFP, DESC, SeV	0.711	0.850	0.855	0.861	0.841	0.869	0.927
M4-2	MGF, MFP, DESC, FPeV	0.719	0.851	**0**.**860**	0.869	0.853	0.867	0.929
M4-3	MGF, MFP, SeV, FPeV	0.705	0.846	0.852	0.859	0.846	0.859	0.921
M4-4	MGF, DESC, SeV, FPeV	0.707	0.849	0.853	0.859	0.841	0.865	0.926
M4-5	MFP, DESC, SeV, FPV	**0**.**720**	0.849	**0**.**860**	**0**.**871**	0.856	0.864	**0**.**930**
M5-1	MGF, DESC, SeV, FPeV, MFP	0.717	0.853	0.858	0.864	0.850	0.867	0.925

Standard deviation value for each split for the above table is given in Additional file 1: S4

Highest values in each metric is given in bold

It can be seen from Table 3 that meta features in M3 and M4 show overall better performance for most of the metrics. In the M4 meta-feature category, M4-5 achieves the best results of MCC: 0.720, ACC: 0.860, PPV: 0.871 and AUC: 0.930. In the M3 meta-feature category, M3-2 achieves the best results for NPV: 0.855 and SEN: 0.874. M3-5 also achieves similar performance of 0.874 for SEN to that of M3-2. Similarly for AUC, M3-7 achieves a similar performance of 0.930 compared to that of M4-5. For SPE however, none of the base-feature combinations (ranging from M2 to M5) improves the performance over M1-1 which is 0.868. Interestingly for SPE, the individual lower performance of MGF, FPeV and SeV (M1-2: 0.792, M1-4: 0.795 and M1-5: 0.791) is improved substantially with meta features comprised of any of the combinations (M2-3: 0.830, M2-4: 0.833 and M2-10: 0.835). This improvement offers some perspective on potentially better ensembling performance even if the individual performance is relatively lower for MGF, FPeV and SeV.

### Effectiveness of meta features

**Fig. 4:**
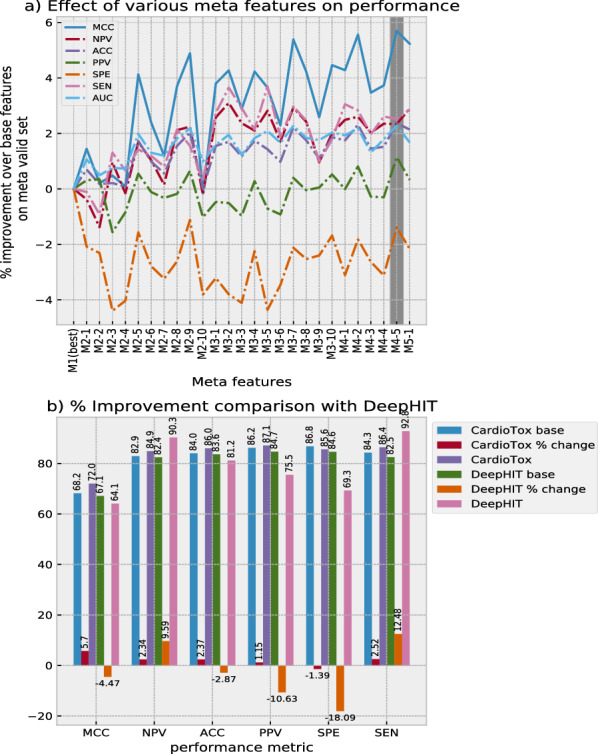
**a** shows the affect of various meta features in terms of % improvement over the base features using an ensemble stage of CardioTox framework on meta valid set. **b** shows the % difference of CardioTox and DeepHIT from their respective best base models performance for various performances metrics

In order to investigate the effectiveness of meta features (M2–M5) as compared to the ones which use only single individual base features (M1), we computed % improvement of each of the meta feature ranging from M2 to M4 over best M1 on the meta validation set as shown in Fig. 4a. An overall improvement can be observed in MCC, NPV, ACC, SEN and AUC. For PPV, more fluctuations across zero axis are observed for various meta features. For SPE, there is overall decrease in performance with relatively bigger fluctuations on the negative side. It can be observed from Fig. 4a and Table 3 that for meta feature M4-5, 4 out of 7 metrics shows improvement as compared to best M1. Thus we select meta feature M4-5 as the final unique combination of base features for our CardioTox net framework for further analysis and final evaluation against external test sets.

In Figure 4b, we show the % difference of CardioTox and DeepHIT from their respective best base model performances for various performance metrics. The values in Fig. 4b are retrieved from Table 2 of the DeepHIT publication [19] and Table 3 for CardioTox. As shown in Table 2 of DeepHIT, the best performance is shown by Descriptor-based DNN for all metrics. DeepHIT is optimized for SEN and NPV with a substantial sacrifice of MCC, ACC, PPV and SPE. It improves SEN by 12.48% and NPV by 9.59% with a sacrifice of 4.47% MCC, 2.87% ACC, 10.63% PPV and 18.09% SPE. On the other hand, CardioTox net improves MCC by 5.7%, NPV by 2.34%, ACC by 2.37%, PPV by 1.15% and SEN by 2.52% with a sacrifice of 1.39% in SPE only. With an overall improvement in nearly all the metrics for a relatively little sacrifice of SPE as compared to DeepHIT, CardioTox net performance can be considered more robust.

### Comparative landscape using the external independent test sets

We compared CardioTox net results with state of the art methods such as DeepHIT [19], CardPred [8], OCHM Predictor-I and OCHM Predictor-II [11] and Pred-hERG 4.2 [18] on three external test sets given in Table 4. For test set-I and test set-II, CardioTox net achieves improved performance for all metrics except SEN where its performance is the same as achieved by second best method DeepHIT. The achieved performance for MCC is (0.599, 0.452), PPV is (0.893, 0.455) and SPE is (0.786, 0.600) over DeepHIT for test set-I and test set-II respectively. The SEN is 0.833 for test set-I and 0.909 for test set-II which is the same as achieved by DeepHIT. For ACC and NPV, the performance for test set-I and test set- II is (0.810, 0.755), and (0.688, 0.947) which is also better than DeepHIT. OCHM-Predictor I, II achieves better performance for PPV and SPE but lags behind significantly in all other metrics for both test sets. Pred-hERG 4.2 performs reasonably well for SEN in both tests but performs worse in other metrics. Interestingly for test-set II, OCHEM-Predictor I and II performs reasonably well for PPV and SPE with less sacrifice in other metrics as compared to its performance on test set-I. For test set-III which is relatively larger, our method achieves better performance for all metrics as compared to DeepHIT except SEN where it achieves same performance as DeepHIT. For test set-III as well, OCHEM Predictor-I achieves better performance for PPV and SPE only while legging behind significantly in other metrics. For SEN though, Pred-hERG 4.2 achieves the highest value.

**Table 4:** Comparison of CardioTox with other methods using three external independent test sets. B-ACC refers to balanced accuracy

Evaluation data	Methods	MCC	NPV	ACC	PPV	SPE	SEN	B-ACC
Test set-I	CardioTox	**0**.**599**	**0**.**688**	**0**.**810**	0.893	0.786	**0**.**833**	**0**.**810**
	DeepHIT	0.476	0.643	0.773	0.833	0.643	0.833	0.738
	CardPred	0.193	0.421	0.614	0.760	0.571	0.633	0.602
	OCHEM Predictor-I	0.149	0.333	0.364	**1**.**000**	**1**.**000**	0.067	0.534
	OCHEM Predictor-II	0.164	0.351	0.432	0.857	0.929	0.200	0.564
	Pred-hERG 4.2	0.306	0.538	0.705	0.774	0.500	0.800	0.650
	Random Forest	0.092	0.375	0.547	0.714	0.428	0.666	0.547
	Support Vector Machines	0.00	0.318	0.5	NA	1.0	0.0	0.500
	Gradient Boosting	0.133	0.375	0.571	0.750	0.642	0.500	0.571
Test set-II	CardioTox	**0**.**452**	**0**.**947**	**0**.**755**	0.455	0.600	**0**.**909**	**0**.**754**
	DeepHIT	0.398	0.941	0.721	0.417	0.533	0.909	0.721
	CardPred	0.049	0.750	0.527	0.294	0.600	0.454	0.527
	OCHEM Predictor-I	0.372	0.800	0.648	**0**.**666**	**0**.**933**	0.364	0.648
	OCHEM Predictor-II	0.310	0.794	0.632	0.571	0.900	0.364	0.632
	Pred-hERG 4.2	0.146	0.813	0.580	0.320	0.433	0.727	0.580
	Random Forest	0.397	0.941	0.721	0.416	0.533	0.909	0.721
	Support Vector Machines	0.345	0.933	0.683	0.384	0.466	0.909	0.687
	Gradient Boosting	0.421	0.941	0.727	0.423	0.550	0.909	0.729
Test set-III	CardioTox	**0**.**220**	**0**.**986**	**0**.**746**	0.113	0.698	0.794	**0**.**746**
	DeepHIT	0.134	0.981	0.660	0.074	0.526	0.794	0.660
	CardPred	0.112	0.975	0.633	0.072	0.561	0.705	0.633
	OCHEM Predictor-I	0.162	0.959	0.562	**0**.**25**	**0**.**978**	0.147	0.562
	OCHEM Predictor-II	0.108	0.961	0.575	0.117	0.915	0.235	0.575
	Pred-hERG 4.2	0.151	0.986	0.680	0.077	0.508	0.852	0.680
	Random Forest	0.179	0.985	0.714	0.085	0.546	0.882	0.714
	Support Vector Machines	0.147	0.981	0.668	0.068	0.365	**0**.**970**	0.667
	Gradient Boosting	0.182	0.981	0.701	0.083	0.551	0.882	0.716

Highest values across each metric and test set is given in bold

DeepHIT is specifically designed and trained to obtain better NPV and SEN by using physicochemical descriptors, fingerprints and graph features with three deep learning base models. CardPred used an individual neural network model (out of six other models) with physicochemical descriptors and fingerprints. OCHMI and OCHMII used range of machine learning models trained on various types of high level physicochemical descriptors. Pred-hERG 4.2 used fingerprints and molecular descriptors with support vector machines to classify the molecules for hERG blocking activity. By using a step-wise training strategy with base and meta ensemble models, CardioTox net shows robust performance against a range of accuracy metrics as compared to the state of the art methods on three independent test sets.

We also compared our results with three classical machine learning methods such as random forest [53], support vector machines [54] and gradient boosting algorithm [55] as shown in Table 4. We first converted all SMILES training as well as test data into 995 2D and 3D physicochemical descriptors (DESC) using Mordred [34]. For all of the three classical methods, we used scikit-learn [56] machine learning library with default settings. For the test set-I which has more positive samples, all three classical machine learning performs the worst of all other methods in nearly all metrics. Support vector machines performs randomly for test set-I. Random forest and gradient boosting performs slightly better than a random classifier. For test set-II and III which have more negative samples, classical methods performance is comparable to other deep learning based methods as shown in Table 4. It should be noted that our model assigns a probability to each molecule under test. The value of the probability if greater than or equal to 0.5 declares the molecule to be hERG blocker.

## Conclusion

In this study, we introduced a deep learning based framework called CardioTox net for classifying drug-like molecules as hERG blockers and hERG non blockers. Our approach is based on step-wise training of base and meta ensemble deep learning models. In the first step, 5 deep learning base models are trained and validated. Each of these base models use different types of base features ranging from high level to low level descriptors and their variants. In the second step of training, the output of base models is concatenated to form meta features for training and validating the meta ensemble model. We found that high level physicochemical, low level fingerprints, SMILES embedding vectors and fingerprint embedding vectors when used to create meta features for the meta ensemble model, enhance the performance over a wide range of metrics for the cardio toxicity prediction task. We evaluated our framework against various classification metrics using three independent test sets and obtained a robust performance compared to state of the art methods. Our framework is a robust method for classifying small drug-like molecules as hERG blockers and hERG non blockers.

## Supplementary Information

**Additional file 1: **Data preparation, SMILES embedding vectors procedure, standard deviation for base and meta features validation.

**Additional file 2: ** List of molecular descriptors used for the development of the descriptor-based FCNND. Information on atom descriptors used for the development of the graph-based GCNN model.

**Additional file 3: **Top 3 similar molecules in training data for each molecule of all three test sets.

## Data Availability

Data: train and test data which is used in our method is as follows. Train data: The training data used in this study can be found at https://github.com/Abdulk084/CardioTox/blob/master/data/train_validation_cardio_tox_data.tar.xz. Test set-I: The positively biased test set can be found at https://github.com/Abdulk084/CardioTox/blob/master/data/external_test_set_pos.csv. Test set-II: The negatively biased test set can be found at https://github.com/Abdulk084/CardioTox/blob/master/data/external_test_set_neg.csv. Test set-III: Relatively larger negatively biased test set can be found at https://github.com/Abdulk084/CardioTox/blob/master/data/external_test_set_new.csv.

## Code availability

Code for our method is available on our GitHub repository as follows. Name: CardioTox net. Home page: https://github.com/Abdulk084/CardioTox. Operating system: Ubuntu 20.04. Programming language: Python 3.7.7.
