# Supplementary material for: CardioTox net: a robust predictor for hERG channel blockade based on deep learning meta-feature ensembles
Source: J Cheminform. 2021 Aug 16;13:60. doi: 10.1186/s13321-021-00541-z (PMC8365955; doi:10.1186/s13321-021-00541-z)
Supplement: Supplementary file 1 — Additional file 1: Data preparation, SMILES embedding vectors procedure, standard deviation for base and meta features validation. [file 13321_2021_541_MOESM1_ESM.pdf]

Supplementary material S1, S2, S3 and S4 for CardioTox

Abdul Karim et al.

August 9, 2021

1 S1: Data Preparation

A dataset consisting of molecular structures labelled as hERG and non-hERG blockers in the form of SMILES strings was obtained from the DeepHIT authors[1] and was curated from five sources, the BindingDB database (3056 hERG blockers, 3039 hERG non-blockers) [2], ChEMBL bioactivity database (4859 hERG blockers, 4751 hERG non-blockers) [3], and literature derived (4355 hERG blockers, 3534 hERG non-blockers) [4], (1545 hERG blockers, 816 hERG non-blockers) [5], (2849 hERG blockers, 1202 hERG non-blockers) [6]. SMILES strings from all the 5 sources were standardized using using RDkit `http://www.rdkit.org/` and MolVS `https://molvs.readthedocs.io/en/latest/` as described by Ryu et al [1] and shown in Figs S1 a. After standardization, each data source was split into four sets such as 70% base training set, 10% base validation set, 10% meta training set and 10% meta validation set. All redundant molecules were removed and respective sets were merged together to form a combined base training set, base validation set, meta training set and meta validation set. We used test set-I from DeepHIT "as is" which contains more hERG blockers than non-blockers. Pairwise Tanimoto similarity [1] was computed between all molecules of combined data sets with those of molecules in test set-I obtained from DeepHIT. All those molecules in the combined data sets, the Tanimoto similarity of which are >0.7 to any of the molecule in test set-I were removed, thus forming a gold standard training and validation data as shown in Figs S1 a.

In order to evaluate our model on another independent test set which should contain more non blockers molecules, we curated 110 hERG blockers and 336 hERG non-blockers from "E3 training" set of Siramshetty at al. [7]. The reason we curated from E3 training is because it contains molecules with potency threshold (IC_{50}) values < 10 µM considered to be hERG blockers and (IC_{50}) values ≥ 10 µM considered to be hERG non-blockers which is compatible with other datasets used in our study. Besides, E3 training is also negatively imbalanced which contains more non-blockers than blocker molecules, as test set-II is aimed to be negatively imbalanced unlike test set-I which is positively imbalanced. We also obtained 9250 molecules from Kumar et al. [8] with pIC_{50}

values as potency threshold. We converted the unit of potency from pIC_{50} to IC_{50} and labelled molecules with (IC_{50}) values < 10 µM as hERG blockers and (IC_{50}) values ≥ 10 µM as hERG non-blockers. Both data sets were merged together and all those molecules with Tanimoto similarity > 0.7 to any molecule in gold standard data training and validation or test set-I were removed. Thus we obtained test set-II which contains more non blocker molecule than blockers and is dissimilar to both gold standard training and validation as well as test set-I as shown in Figs S1 b. Both test set-I and II are relatively small in number, so we curated another larger independent test set from very recent work of Siramshetty at al. [9]. This larger test set is also negatively biased with 53 blockers and 786 non-blockers. All molecules were compared with training set, test set-I and test set-II in terms of pairwise tanimoto similarity. Molecules with tanimoto similarity > 0.7 to any of the molecules in training set, test set-I or test set-II were removed to form test set-III. Thus we obtain total of 706 hERG non-blockers and 34 hERG blockers in the test set-III as shown in Figs S1 b.

2 S2: SMILES embedding vectors

Based on the training data, SMILES vocabulary is generated using tokenizer module developed by Reverie Labs, the link of which is given below.

https://blog.reverielabs.com/transformers-for-drug-discovery/.

Each SMILES string is converted into fixed size numerical vector based on mapping dictionary of SMILES vocab as shown in Figs S2. The mapping dictionary maps each SMILES vocab element to a numerical value. The length of the longest SMILES string is 97 in terms of SMILES vocab element in the training data considered for this work.

3 S3: Standard deviation for base features validation

Tables S1 shows standard division for each split of base validation set in training the individual base models.

Tables S1: Standard deviation values for 10 fold cross validated performance of the base models in individual prediction stage on base validation set using their respective base features.

Base features	MCC	NPV	ACC	PPV	SPE	SEN	AUC
DESC	0.024	0.019	0.012	0.021	0.025	0.028	0.011
MGF	0.020	0.022	0.010	0.025	0.034	0.024	0.006
MFP	0.016	0.023	0.008	0.026	0.035	0.027	0.007
MFP	0.016	0.031	0.008	0.026	0.042	0.038	0.007
FPeV	0.024	0.028	0.012	0.024	0.040	0.034	0.010
SeV	0.018	0.021	0.009	0.019	0.036	0.037	0.007

4 S4: Standard deviation for meta features validation

Tables S2 shows standard division for each split of meta validation set in 10 fold validation process.

Tables S2: Standard deviation values for 10 fold cross validation results for various meta features on meta validation set.

Meta Features	Base features	MCC	NPV	ACC	PPV	SPE	SEN	AUC
M1-1	DESC, DESC	0.022	0.013	0.011	0.014	0.017	0.025	0.008
M1-2	MGF, MGF	0.023	0.018	0.012	0.020	0.034	0.022	0.008
M1-3	MFP, MFP	0.021	0.023	0.011	0.025	0.038	0.030	0.008
M1-4	FPeV, FPeV	0.034	0.025	0.018	0.028	0.044	0.028	0.011
M1-5	SeV, SeV	0.019	0.025	0.010	0.025	0.042	0.031	0.007
M2-1	MGF, MFP	0.019	0.015	0.009	0.014	0.017	0.012	0.007
M2-2	MGF, DESC	0.019	0.015	0.009	0.014	0.017	0.012	0.007
M2-3	MGF, SeV	0.015	0.019	0.008	0.020	0.025	0.023	0.005
M2-4	MGF, FPeV	0.019	0.013	0.010	0.021	0.029	0.017	0.004
M2-5	MFP, DESC	0.014	0.012	0.007	0.016	0.022	0.015	0.007
M2-6	MFP, SeV	0.025	0.017	0.012	0.017	0.032	0.020	0.006
M2-7	MFP, FPeV	0.024	0.023	0.012	0.018	0.023	0.021	0.006
M2-8	DESC, SeV	0.020	0.021	0.010	0.020	0.024	0.021	0.005
M2-9	DESC, FPeV	0.023	0.018	0.011	0.021	0.027	0.023	0.009
M2-10	SeV, FPeV	0.019	0.013	0.009	0.012	0.020	0.012	0.005
M3-1	MGF, MFP, DESC	0.017	0.014	0.009	0.011	0.019	0.017	0.006
M3-2	MGF, MFP, SeV	0.023	0.019	0.012	0.025	0.032	0.022	0.006
M3-3	MGF, MFP, FPeV	0.025	0.019	0.013	0.021	0.027	0.017	0.009
M3-4	MGF, DESC, SeV	0.021	0.026	0.011	0.023	0.029	0.029	0.008
M3-5	MGF, DESC, FPeV	0.015	0.014	0.007	0.009	0.022	0.016	0.009
M3-6	MGF, SeV, FPeV	0.016	0.013	0.008	0.018	0.019	0.009	0.008
M3-7	MFP, DESC, SeV	0.014	0.027	0.006	0.020	0.024	0.027	0.006
M3-8	MFP, DESC, FPeV	0.009	0.018	0.004	0.013	0.015	0.019	0.005
M3-9	MFP, SeV, FPeV	0.008	0.018	0.004	0.015	0.023	0.022	0.004
M3-10	DESC, SeV, FPeV	0.021	0.026	0.010	0.012	0.017	0.028	0.007
M4-1	MGF, MFP, DESC, SeV	0.021	0.022	0.010	0.017	0.023	0.021	0.008
M4-2	MGF, MFP, DESC, FPeV	0.025	0.021	0.012	0.020	0.024	0.020	0.007
M4-3	MGF, MFP, SeV, FPeV	0.020	0.017	0.010	0.016	0.020	0.018	0.009
M4-4	MGF, DESC, SeV, FPeV	0.012	0.017	0.006	0.015	0.023	0.020	0.005
M4-5	MFP, DESC, SeV, FPV	0.020	0.015	0.010	0.013	0.017	0.017	0.006
M5-1	MGF, DESC, SeV, FPeV, MFP	0.021	0.017	0.010	0.015	0.021	0.020	0.008

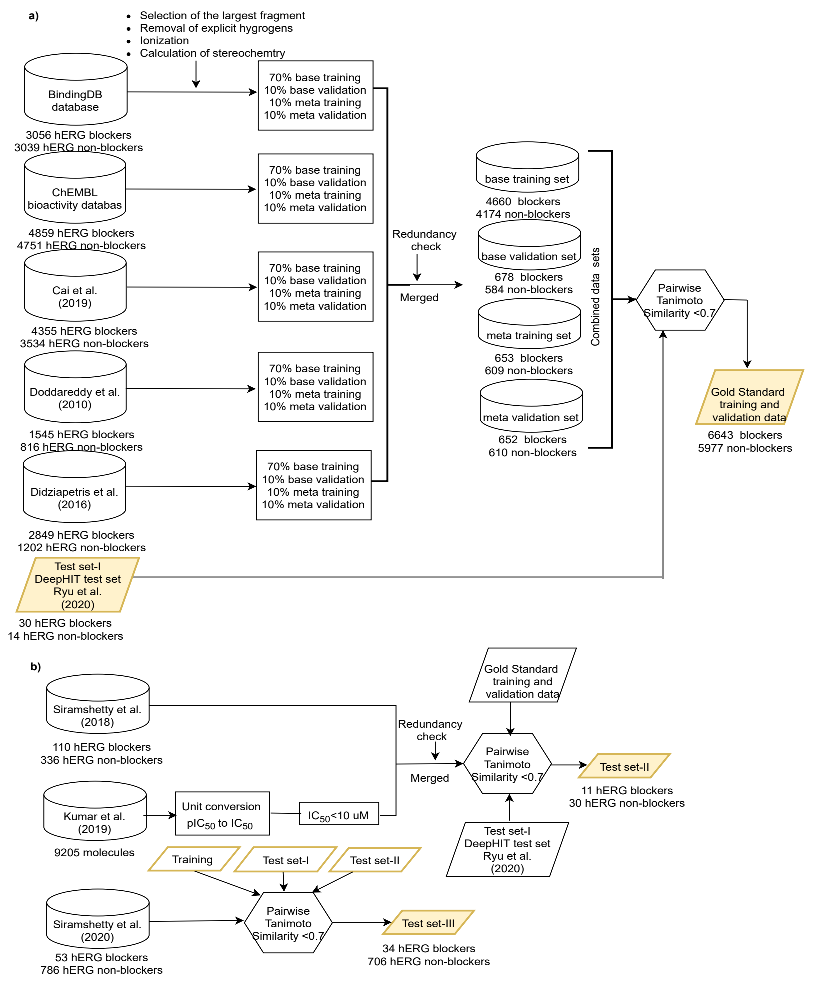

Figs S1: a) Preparation of gold standard training data as per the procedure described in DeepHIT. b) Preparation of external independent test set-II, test set-III.

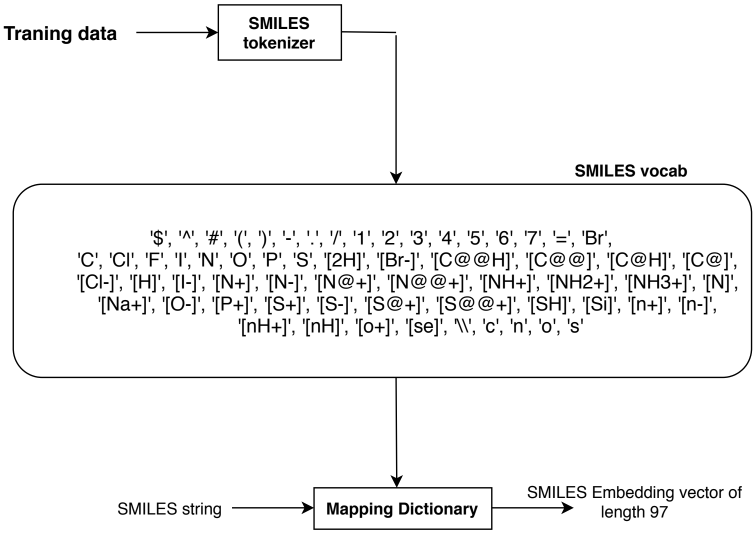

Figs S2: SMILES embedding vectors based on the vocab elements.

References

[1] Jae Yong Ryu, Mi Young Lee, Jeong Hyun Lee, Byung Ho Lee, and Kwang-Seok Oh. Deephit: a deep learning framework for prediction of herg-induced cardiotoxicity. *Bioinformatics*, 36(10):3049–3055, 2020.

[2] Michael K Gilson, Tiqing Liu, Michael Baitaluk, George Nicola, Linda Hwang, and Jenny Chong. Bindingdb in 2015: a public database for medicinal chemistry, computational chemistry and systems pharmacology. *Nucleic acids research*, 44(D1):D1045–D1053, 2016.

[3] Anna Gaulton, Louisa J Bellis, A Patricia Bento, Jon Chambers, Mark Davies, Anne Hersey, Yvonne Light, Shaun McGlinchey, David Michalovich, Bissan Al-Lazikani, et al. Chembl: a large-scale bioactivity database for drug discovery. *Nucleic acids research*, 40(D1):D1100–D1107, 2012.

[4] Chuipu Cai, Pengfei Guo, Yadi Zhou, Jingwei Zhou, Qi Wang, Fengxue Zhang, Jiansong Fang, and Feixiong Cheng. Deep learning-based prediction of drug-induced cardiotoxicity. *Journal of chemical information and modeling*, 59(3):1073–1084, 2019.

[5] Munikumar R Doddareddy, Elisabeth C Klaasse, Adriaan P IJzerman, and Andreas Bender. Prospective validation of a comprehensive in silico herg model and its applications to commercial compound and drug databases. *ChemMedChem*, 5(5):716–729, 2010.

[6] Remigijus Didziapetris and Kiril Lanevskij. Compilation and physicochemical classification analysis of a diverse herg inhibition database. *Journal of computer-aided molecular design*, 30(12):1175–1188, 2016.

[7] Vishal B Siramshetty, Qiaofeng Chen, Prashanth Devarakonda, and Robert Preissner. The catch-22 of predicting herg blockade using publicly accessible bioactivity data. *Journal of Chemical Information and Modeling*, 58(6):1224–1233, 2018.

[8] Leela Sarath Kumar Konda, S Keerthi Praba, and Rajendra Kristam. herg liability classification models using machine learning techniques. *Computational Toxicology*, 12:100089, 2019.

[9] Vishal B Siramshetty, Dac-Trung Nguyen, Natalia J Martinez, Noel T Southall, Anton Simeonov, and Alexey V Zakharov. Critical assessment of artificial intelligence methods for prediction of herg channel inhibition in the "big data" era. *Journal of Chemical Information and Modeling*, 60(12):6007–6019, 2020.
